# Therapeutic potential of microRNA-506 in cancer treatment: mechanisms and therapeutic implications

**DOI:** 10.3389/fonc.2025.1524763

**Published:** 2025-04-03

**Authors:** Shuzhen Mao, Junyan Li, Jiahui Huang, Lili Lv, Qilian Zhang, Qing Cheng, Xiaojing Liu, Zhiwei Bi, Jing Yao

**Affiliations:** ^1^ Department of Pharmacy, Shandong First Medical University and Shandong Academy of Medical Sciences, Jinan, Shandong, China; ^2^ Department of Pathology, Second People’s Hospital of Ningyang, Taian, Shandong, China; ^3^ Jining Key Laboratory of Pharmacology, School of Basic Medicine, Jining Medical University, Jining, Shandong, China; ^4^ Department of Pathology, People’s Hospital Affiliated to Shandong First Medical University, Jinan, Shandong, China

**Keywords:** cancer, microRNA-506, circular RNAs, long non-coding RNAs, drug resistance

## Abstract

Cancer is a complex and highly lethal disease marked by unchecked cell proliferation, aggressive behavior, and a strong tendency to metastasize. Despite significant advancements in cancer diagnosis and treatment, challenges such as early detection difficulties, drug resistance, and adverse effects of radiotherapy or chemotherapy continue to threaten patient survival. MicroRNAs (miRNAs) have emerged as critical regulators in cancer biology, with miR-506 being extensively studied and recognized for its tumor-suppressive effects across multiple cancer types. This review examines the regulatory mechanisms of miR-506 in common cancers, focusing on its role in the competing endogenous RNA (ceRNA) network and its effects on cancer cell proliferation, apoptosis, and migration. We also discuss the potential of miR-506 as a therapeutic target and its role in overcoming drug resistance in cancer treatment. Overall, these insights underscore the therapeutic potential of miR-506 and its promise in developing novel cancer therapies.

## 1 Introduction

Cancer is a terrible disease, and cancer cells usually proliferate in an uncontrolled manner. Cancer cells demonstrate invasiveness and metastasis during proliferation, leading to serious consequences (1, 2). As the second leading cause of death globally, cancer treatment has always posed an enormous challenge. Current approaches to cancer management include surgery, chemotherapy, radiation therapy, targeted therapies, and their combinations of these. However, despite advancements in traditional treatment approaches and the emergence of new treatments, the cancer-related mortality rate continues to increase (3, 4). The main challenges currently faced in cancer treatment include difficulties in detecting and diagnosing early-stage cancer, susceptibility to metastasis, increased drug resistance, and individual variations during treatment. Therefore, actively exploring new approaches to the diagnosis and management of cancer is of great importance.

MicroRNAs (miRNAs) are a class of endogenous, highly conserved, non-coding single-stranded RNA molecules, typically consisting of 18 to 25 nucleotides in length (5, 6). They primarily function to regulate the stability of mRNA for protein-coding genes negatively. In most cases, miRNAs promote the degradation of target mRNA or reduce its proper translation initiation by binding to complementary sequences within the 3′ untranslated region (UTR) (4). The discovery of the first miRNA, lin-4, in 1993 unveiled a novel mechanism of gene regulation (7). An increasing body of research has demonstrated that dysregulation of miRNAs is closely associated with the onset and progression of various human diseases (8, 9), especially in cancer (10). miRNAs have now been widely recognized as tumor suppressors or oncogenes in numerous human malignancies, playing pivotal roles in cancer development and progression by regulating multiple signaling pathways and cellular functions (10, 11).

MicroRNA-506 (miR-506) is located on the q27.3 locus of the X chromosome (chrXq27.3) (12). This locus houses a key miRNA cluster containing 30 mature miRNAs, which are further categorized into two groups according to their genomic location: miR-506-514 and miR-888-892, with miR-506 being the most well-known member of this cluster (13). According to the source, miR-506 is classified as 5p or 3p, with miR-506-3p holding a prominent place in miR-506 (14). Recent preclinical studies have highlighted the tumor-suppressive potential of miR-506 across multiple cancer types, positioning it as a promising candidate for clinical translation but occasionally displaying oncogenic properties depending on the cancer type and molecular targets. miR-506 inhibits hepatocellular carcinoma (HCC) cell proliferation and tumorigenicity by targeting Rho-associated protein kinase 1 (ROCK1), which is upregulated in HCC tissues and inversely correlated with miR-506 levels, supporting miR-506 mimics as a therapeutic strategy (15). miR-506 suppresses Non-Small Cell Lung Cancer (NSCLC) progression by regulating Tubby-like protein 3 (TULP3), inducing mitochondrial apoptosis, and *in vivo* studies confirm its ability to reduce tumor growth, highlighting its role in NSCLC therapy (16). miR-506-3p targets MTMR6, inhibiting cell proliferation and promoting apoptosis. Its downregulation in ovarian tumors underscores its potential as a therapeutic target (17). Conversely, in colorectal cancer (CRC), miR-506 inhibits NR4A1, an orphan nuclear receptor driving oncogenic signaling (18). In breast cancer (BC), miR-506 modulates the specificity protein (SP)1/SP3 to demethylate the tumor-suppressive lncRNA MEG3, thereby inhibiting metastasis (19). These distinct targets underscore the need for cancer-specific mechanistic studies. While miR-506 is often recognized for its tumor-suppressive functions, limited evidence suggests it can also exhibit pro-cancer effects in specific tumor types. In melanoma, miR-506 has been identified as part of the miRNA-506-514 cluster, critical in initiating melanocyte transformation and promoting tumor growth. The overexpression of miR-506 in melanoma models has been linked to enhanced tumorigenicity, suggesting its potential as an oncogenic driver in this context (14). miRNA-506 expression is elevated in the plasma of esophageal squamous cell carcinoma (ESCC) patients compared to healthy individuals. It correlates with disease severity indicators, which exhibit diagnostic significance for ESCC and are associated with patient prognosis, suggesting its potential as a molecular marker for both diagnosis and outcome prediction in ESCC (20). Compared to miR-506-3p, miR-506-5p has been investigated in only a limited number of cancer types, including gastric cancer (GC) and glioma (21–24).

Despite the increasing research on miR-506 due to its recognized importance in cancer regulation, several unmet needs remain in its therapeutic application. The expression pattern and function of miR-506 are complex and, at times, contradictory, highlighting its distinct role across different cancer types. Growing evidence suggests that miR-506 primarily functions as a tumor suppressor. However, miR-506 exhibits oncogenic properties in specific contexts, depending on the cancer type and cellular environment. For instance, miR-506 promotes tumorigenesis in uveal melanoma (UM) (25) and contributes to chemoresistance in CC (26). Furthermore, tumor heterogeneity and microenvironmental factors, such as metabolic reprogramming, immune modulation, and its interactions with other non-coding RNAs, such as those involved in competing endogenous RNA (ceRNA) network, further modulate miR-506’s role in cancer progression (17). This functional paradox complicates the development of miR-506-based therapies, as their therapeutic efficacy varies across cancer types and microenvironments. miR-506 simultaneously targets multiple mRNAs, exerting broad regulatory effects across multiple genes (27). This characteristic makes miR-506 a promising candidate for treating multifactorial diseases such as cancer. However, its extensive regulatory activity also increases the risk of off-target effects, potentially disrupting normal cellular function (28). Due to its inherent instability and poor tissue specificity, effective delivery remains a significant challenge. Naked miR-506 molecules undergo rapid degradation in circulation, reducing their bioavailability and limiting their therapeutic potential (29). Exosome- and nanoparticle-based miRNA delivery systems have shown potential in enhancing miRNA stability and targeting specificity (30, 31). Despite these advancements, optimized delivery strategies are still needed to ensure safe and effective tumor-specific uptake while minimizing toxicity. The clinical validation of miR-506-based therapies remains limited, necessitating further large-scale, multicenter clinical studies to assess their safety, efficacy, and potential integration into standard cancer treatments (32).

This review distinguishes itself from previous studies by providing a comprehensive and up-to-date perspective on the role miR-506 in cancer therapy. Unlike previous reviews that primarily focused on its tumor-suppressive function, this review systematically examines the oncogenic and tumor-suppressive roles of miR-506, offering insights into the underlying mechanisms contributing to this paradox. Furthermore, this review highlights the influence of the ceRNA network on miR-506 function, an aspect that has not been extensively explored in previous studies. This review provides a detailed analysis of the molecular mechanisms through which miR-506 modulates drug resistance. Additionally, it discusses the challenges associated with the development of miRNA-based therapies. It aims to elucidate the potential of the miR-506 family as a therapeutic target in cancer, thereby advancing the development of more effective and safer cancer treatment strategies.

## 2 ceRNA network regulated by miR-506 in cancer

The ceRNA network is a relatively novel concept in molecular biology that describes how different RNA species—primarily messenger RNAs (mRNAs), long non-coding RNAs (lncRNAs), and circular RNAs (circRNAs)—interact with miRNAs in a manner that influences gene expression (33). ceRNAs are endogenous transcripts that harbor shared miRNA response elements (MREs) and regulate one another by competing for binding to shared miRNAs, thereby reducing the availability of miRNAs for their target mRNAs (33, 34). Since miRNAs typically function as post-transcriptional repressors by targeting mRNAs for degradation or translational inhibition, ceRNA competition can modulate mRNA stability and translation, ultimately affecting key cellular processes such as proliferation, differentiation, and apoptosis (35).

The interplay between ceRNAs is orchestrated by shared miRNAs, forming a finely balanced regulatory network (33). Dysregulation of ceRNA networks in cancer is often driven by genetic mutations, epigenetic alterations, or aberrant RNA expression, disrupting critical oncogenes and tumor suppressors (36). Increasing evidence suggests that ceRNAs play a crucial role in tumorigenesis by modulating the expression of oncogenes and tumor suppressors through miRNA-mediated interactions (37). Functionally, ceRNAs in cancer can be categorized into the following roles (34) (1): regulation of cancer cell proliferation, migration, and invasion. For instance, TINCR functions as a ceRNA by sequestering miR-544a from its target gene FBXW7, thereby suppressing lung cancer cell proliferation and invasion (38). Conversely, circ-TLK1 and lncRNA HOSD-AS1 enhance HCC progression by sponging miR-138-5p and miR-130a-3p, respectively, leading to upregulation of SOX4, a key oncogene that promotes proliferation, invasion, and metastasis (39) (2). modulation of epithelial-mesenchymal transition (EMT). lncRNA CCAT1 functions as a ceRNA to promote EMT in gliomas and retinoblastomas, thereby facilitating tumor progression (40, 41). In contrast, lncRNA CASC2 acts as a tumor suppressor by inhibiting HCC cell migration, invasion, and EMT progression through miRNA-mediated regulation (42) (3). promotion or suppression of tumor initiation. RSU1P2, for example, promotes cervical cancer development by competitively binding to the shared microRNA let-7a, thereby enhancing oncogene expression (43). In contrast, circRNA Cir-ITCH exerts tumor-suppressive effects by functioning as a ceRNA to inhibit bladder cancer progression (44). (4) regulation of chemotherapeutic sensitivity and resistance. lncRNA CCAT1 enhances HCC cell proliferation and reduces oxaliplatin-induced apoptosis, thereby contributing to chemoresistance (45). Conversely, lncRNA CASC2 increases the sensitivity of prostate cancer cells to docetaxel, highlighting its potential as a therapeutic target for overcoming drug resistance (46).

Emerging research has revealed that miR-506 not only directly targets mRNAs to regulate cancer progression but also participates in ceRNA networks by interacting with circRNAs and lncRNAs. These interactions create a multifaceted regulatory framework that amplifies miR-506's role in cancer biology (47). Below, we discuss the involvement of circRNAs and lncRNAs in miR-506-mediated ceRNA networks and their implications in cancer.

circRNAs are non-coding RNA molecules distinguished by their strong stability and have recently gained recognition as critical components of gene expression networks (48, 49). circRNAs possess multiple conserved miRNA binding sites and can function as efficient miRNA sponges that specifically adsorb miRNAs, thereby modulating their inhibitory effects on target genes (50). A growing body of evidence supports the notion that circRNAs, functioning as ceRNAs, dominate the regulation of the circRNA-miRNA-mRNA signaling axis (51). For instance, in NSCLC, circRNAFOXO3 (52) and circRNA100565 (53) can inhibit miR-506 by exerting a sponge effect, thereby up-regulating high mobility group box B3 (HMGB3) and high mobility group box A2 (HMGA2) and promoting disease progression. Additionally, has-circRNA0016788 can enhance HCC progression by modulating miR-506-3p and increasing Poly ADP-ribose Polymerase family 14 (PARP14) expression (54). Snail family transcriptional repressor 2 (Snail2) and yes-associated protein 1 (YAP1) are known oncogenes that are up-regulated in cancer (55, 56), circRNAPCNX serves as a sponge for miR-506 and inhibits the anti-cancer activity of miR-506 via the circRNAPCNX-miR-506-Snail2/YAP axis in HCC (57). Another study shows that circRNASYPL1 can down-regulate miR-506 and up-regulate zeste homolog 2 (EZH2) enhancer, facilitating cancer cell metastasis in HCC and exacerbating disease progression (58). As mentioned earlier, studies have shown that miR-506 can also act synergistically with other miRNAs as a target for ceRNA. For example, in CRC, circRNAPACRGL stimulates the growth, invasion, and migration of cancer cells through the miR-142-3p/miR-506-3p-TGF-β1 axis while also influencing neutrophil differentiation from N1 to N2, thereby affecting apoptosis (59).

**Figure 1:**
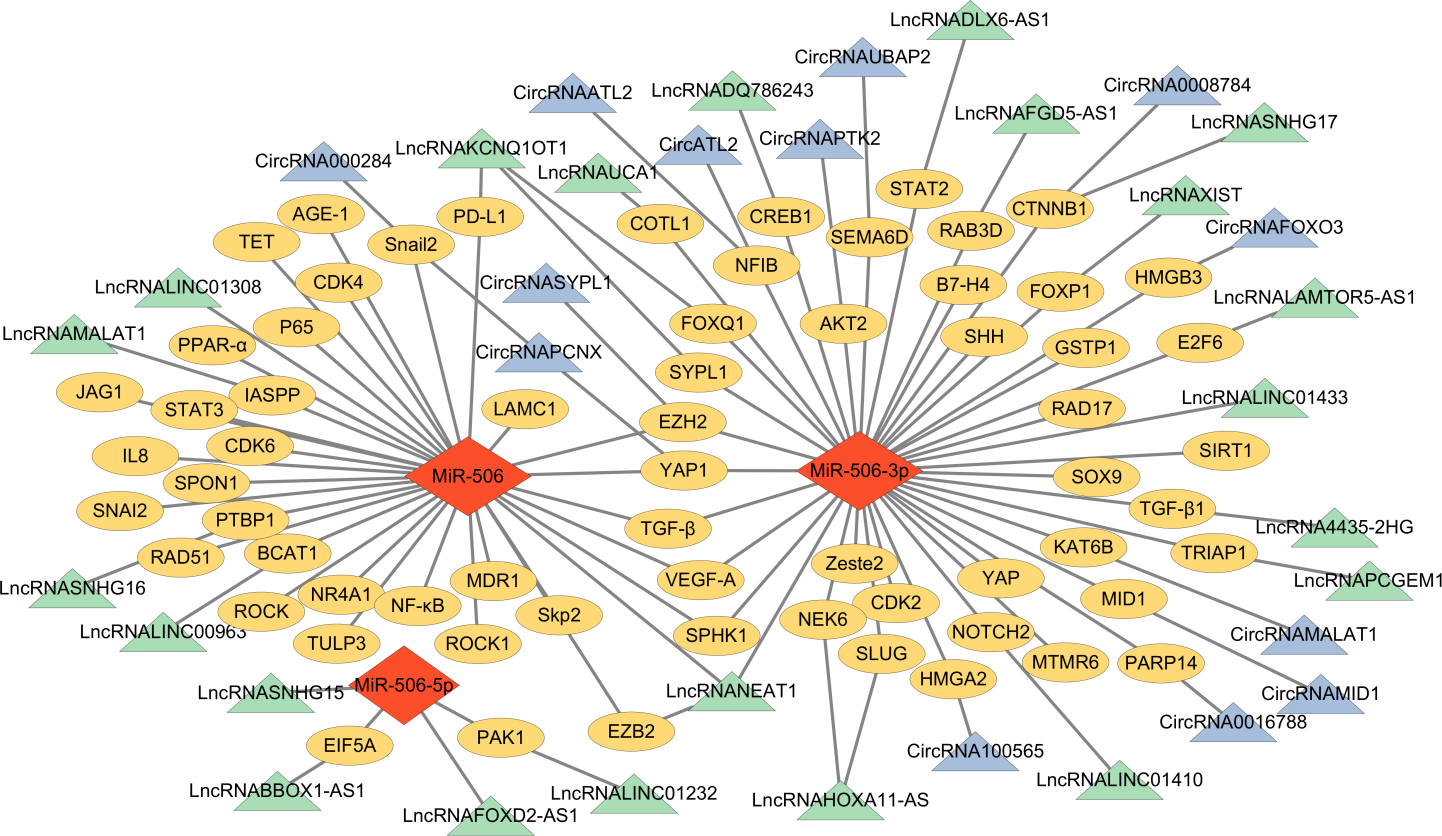
The ceRNA network of miR-506 family members and their target genes. (
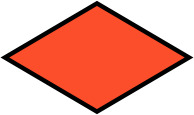
) = miR-506 family members; (
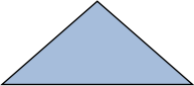
) = circRNAs; (
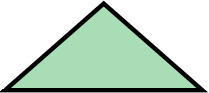
) = lncRNAs; (
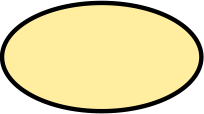
) = target genes.

lncRNAs, similar to circRNAs, have been recognized for their capacity to modulate miR-506 function in cancer through a ceRNA mechanism. lncRNAs, a class of non-coding transcripts exceeding 200 nucleotides in length, account for approximately 60% of the total human transcripts (60). The ongoing advancement of biotechnology in recent years has underscored that lncRNAs play a significant role in regulating cancer-related processes, including tumor cell proliferation, apoptosis, autophagy, EMT, and drug resistance (61). For instance, LINC01433, by targeting miR-506-3p, has been shown to enhance the proliferative, migratory, and invasive properties of nasopharyngeal carcinoma cells (62). In gliomas, lincRNA00963 reduces the expression level of miR-506, thereby affecting the downstream target protein branched-chain Amino Acid Transaminase 1 (BCAT1) and weakening the inhibitory effect of miR-506 on tumors (63). Huang et al. demonstrated through clinical samples and cell experiments that lncRNANEAT1 can exacerbate cancer progression by acting as a sponge for miR-506-3p (64). In summary, within the ceRNA network, circRNAs and lncRNAs typically serve as effective “sponges” for miRNAs, sequestering them from target mRNAs to indirectly regulate gene expression and influence tumor progression, including EMT and drug resistance (65). The relationships among circRNAs, lncRNAs, and miR-506 in different types of cancer are as follows (**Tables 1**, **2**); **Figure 1** shows the ceRNA network of lncRNA or circRNA with miR-506 and corresponding targets in cancer, as well as the direct target of miR-506.

**Table 1:** lncRNA-mediated crosstalk of miR-506.

Cancer type	lncRNA	Expression of miR-506	Target mRNA and its expression	Pathway or axis	Results	References
Nasopharyngeal carcinoma	LINC01433	↓		LINC01433/miR-506-3p axis	promote cancer cell proliferation, migration and invasion	(62)
Non-small lung cancer	LAMTOR5-AS1AS1	↓	E2F6, ↑	LAMTOR5-AS1/miR-506-3p/E2F6 pathway	promote cancer cell proliferation, migration and metastasis suppress cancer cell apoptosis	(188)
	UCA1	↓	COTL1, ↑	UCA1/miR−506−3p/COTL axis	promote cancer cell proliferation, cloning and metastasis suppress cancer cell apoptosis	(68)
Hepatocellular carcinoma	HOXA11-AS	↓	Slug, ↑	HOXA11-AS/miR−506−3p/Slug axis	promote cancer cell proliferation, invasion and EMT	(144)
	KCNQ1OT1	↓	FOXQ1, ↑	KCNQ1OT1/miR-506-3p/FOXQ1 axis	promote cancer cell migration, invasion and EMT	(189)
	MIR4435-2HG	↓	TGFB1, ↑	CXCL1/MIR4435-2HG/miR-506-3p/TGFB1 axis	promote tumorigenesis promote cancer cell migration and invasion	(190)
Osteosarcoma	FGD5-AS1	↓	RAB3D, ↑	FGD5-AS1/ miR-506-3p/RAB3D axis	promote cancer cell proliferation and migration	(191)
Ovarian cancer	XIST	↓	FOXP1, ↑	XIST/miR-506-3p/FOXP1 axis	promote cancer cell proliferation and autophagy suppress cancer cell apoptosis	(192)
	MALAT1	↓	iASPP, ↑	MALAT1/miR-506/IASPP axis	promote cancer cell growth and DNA synthesis	(193)
	DQ786243	↓	CREB1, ↑	DQ786243/miR-506-3p/CREB1 axis	promote cancer cell proliferation, migration, invasion colony formation and wound healing suppress cancer cell apoptosis	(194)
	NEAT1	↓		LIN28B/NEAT1/ miR-506 axis	promote cancer cell proliferation, migration and invasion	(195)
	LINC01308	↓		LINC01308/miR-506 axis	promote cancer cell migration and invasion	(196)
Prostate cancer	PCGEM1	↓	TRIAP1, ↑	PCGEM1/miR-506-3p/TRIAP1 axis	promote cancer cell proliferation, migration and invasion	(197)
Neuroblastoma	DLX6-AS1	↓	STAT2, ↑	DLX6-AS1/miR-506-3p/STAT2 axis	promote cancer cell growth, proliferation, cell cycle and glycolysis	(198)
Glioma	LINC01410	↓	NOTCH2, ↑	MYC/LINC01410 miR-506-3p/ NOTCH2 axis	promote cancer cell proliferation suppress cancer cell apoptosis	(199)
	SNHG17	↓	CTNNB1, ↑	YY1/SNHG17/ miR-506-3p/CTNNB1/Wnt/β-catenin signaling pathway	promote cancer cell growth suppress cancer cell apoptosis	(200)
	LINC00963	↓	BCAT1, ↑	LINC00963/miR-506/BCAT1 axis	promote tumorigenesis promote cancer cell proliferation, cell cycle progression, migration, and invasion	(63)
	FOXD2-AS1	↓	CDK2, cyclinE1, ↑ P21, MMP7, MMP9, ↓	FOXD2-AS1/miR-506-5p axis	promote cancer cell proliferation, migration, metastasis and EMT	(21)
Esophageal squamous cell carcinoma	BBOX1-AS1	↓	EIF5A, ↑	BBOX1-AS1/miR-506-5p/EIF5A/PTCH1/Hedgehog signaling pathway	promote cancer cell proliferation and stemness	(22)
Pancreatic cancer	NEAT1	↓		NEAT1/miR-506-3p axis	promote cancer cell proliferation and cell cycle progression suppress cancer cell apoptosis	(64)
Gastric cancer	SNHG15	↓		SNHG15/miR-506-5p axis	promote cancer cell proliferation, migration, invasion suppress cancer cell apoptosis	(24)
	LINC01232	↓	PAK1, ↑	LINC01232/miR-506-5p/PAK1 axis	promote cancer cell migration, invasion and EMT	(23)
Oral squamous cell carcinoma	Kcnq1ot1	↓	SYPL1, ↑	YY1/Kcnq1ot1/miR-5063p/SYPL1 axis	promote cancer cell viability, colony-forming ability, migration and invasion suppress cancer cell apoptosis	(201)
Retinoblastoma	HOXA11-AS	↓	NEK6, ↑	HOXA11-AS/miR-506-3p/NEK6 axis	promote cancer cell proliferation and cell cycle progression suppress cancer cell apoptosis	(202)

↓, Decrease or downregulation; ↑, Increase or upregulation.

**Table 2:** circRNA-mediated crosstalk of miR-506.

Cancer type	circRNA	Expression of miR-506	Target mRNA and its expression	Pathway or axis	Results	References
Non-small lung cancer	circFOXO3	↓	HMGB3, ↑	circFOXO3/miR-506-3p/HMGB3 axis	promote cancer cell proliferation, migration, and invasion promote tumor growth	(52)
	circ_100565	↓	HMGA2, ↑	circ_100565/miR-506-3p/HMGA2 axis	promote cancer cell proliferation, migration, invasion promotes tumor growth	(53)
Hepatocellular carcinoma	hsa_circ_0016788	↓	PARP14, ↑	hsa_circ_0016788/miR-506-3p/PARP14 axis	promote tumor growth promote cancer cell glycolysis metabolism, cell vitality, proliferation, colony formation, and invasion suppress cancer cell apoptosis	(54)
	circPCNX	↓	PCNX, ↑	circPCNX /miR-506/ PCNX axis	promote cancer cell vitality	(57)
	circSYPL1	↓	EZH2, ↑	CircRNASYPL1/miR-506-3p/EZH2 axis	promote cancer initiation, development progression and invasion	(58)
	circHIPK3	↓	PDK2, ↑	circHIPK3/miR-124 or miR-506/PDK2 axis	promote tumorigenesis promote cancer cell proliferation and invasion	(180)
Colorectal cancer	circPACRGL	↓	TGF-β1, ↑	miR-142-3p or miR-506-3p/TGF-β1 axis	promote cancer cell proliferation, migration, and invasion	(59)
	circPTK2	↓	AKT2, ↑	circPTK2/miR-506-3p/AKT2 axis	promote cancer cell proliferation, migration, and invasion	(203)
	circ-MALAT1	↓	KAT6B, ↑	miR-506-3p/KAT6B axis	promote cancer cell proliferation, migration, and EMT	(204)
Ovarian cancer	circATL2	↓	NFIB, ↑	circATL2/miR-506-3p/NFIB axis	promote the resistance of OC to PTX promote cancer cell colony formation suppress cancer cell apoptosis	(145)
Prostate cancer	circMID1	↓	MID1, ↑	S100A9/circRNAMID1/miR-506-3p/MID1 axis	promote cancer cell proliferation, migration, and invasion	(205)
Cervical cancer	circRNA-000284	↓	Snail2, ↑	circRNA-000284/MiR-506/Snail2 axis	promote cancer cell proliferation and invasion	(206)
Osteosarcoma	circUBAP2	↓	SEMA6D, ↑	circUBAP2/miR-506-3p/SEMA6D axis	promote cancer cell proliferation and invasion promote the resistance of OC to cisplatin suppress cancer cell apoptosis	(207)
Triple-negative breast cancer	circ_0008784	↓	CTNNB1, ↑	circ_0008784/Wnt/β-catenin pathway circ_0008784/miR-506-3p/CTNNB1 axis	promote cancer cell proliferation suppress cancer cell apoptosis	(208)

↓, Decrease or downregulation; ↑, Increase or upregulation.

## 3 Functions of miR-506 in cancer

### 3.1 miR-506 and lung caner

Lung cancer remains the predominant cause of cancer-related mortality globally, with NSCLC accounting for over 85% of cases, and patients with NSCLC are usually diagnosed at advanced stages (66, 67). Decreased levels of miR-506-3p result in enhanced expression of coactosin-like protein 1 (COTL1), thereby promoting the progression of NSCLC (68). Yin et al. demonstrated that miR-506 can promote cancer cell apoptosis, reduce their activity, and achieve anti-cancer effects by participating in oxidative stress and targeting Nuclear factor kappa b (NF-κB) and p65 together (69). The tumor microenvironment affects the disease progression, including the changes in peripheral blood vessels. Vascular endothelial growth factor A (VEGF-A) is a critical cytokine in angiogenesis and is regulated by signal transducer and activator of transcription 3 (STAT3); alginic acid can lower miR-506 levels to inhibit angiogenesis, which is consistent with the results of xenograft experiments (70). Research also indicates that miR-506 can work synergistically with other miRNAs to impede cancer progression. Specifically, combination therapy of miR-143 and miR-506 downregulates the levels of cyclin-dependent kinases (CDK) 1, CDK4, and CDK6 and induces apoptosis in cancer cells, but it does not affect normal lung fibroblasts. This combination therapy can usually inhibit the transition of the cancer cell cycle, equivalent to the effect of clinical cell cycle inhibitors (71); Hossian et al.'s research also confirms this viewpoint (72). In conclusion, these findings demonstrate that miR-506 may function as a tumor suppressor in lung cancer.

### 3.2 miR-506 and liver cancer

Liver cancer (LC) ranks as the fourth most common cause of mortality attributed to cancer, with its incidence showing an upward trend (73). Typically, LC can be broadly categorized into two types: primary liver cancer and metastatic liver cancer. The former encompasses various types, including HCC, intrahepatic cholangiocarcinoma, and mixed liver cancer, with HCC being the most prevalent (74). Research highlights that miR-506-3p can inhibit the growth of LC cells and serve as a biomarker for the diagnosis and prognosis of LC (75). The Hippo signaling pathway is significantly linked to the onset of LC, with YAP critically involved in modulating the response genes within this pathway (76, 77). Researchers found that miR-506-3p can specifically target the 3' UTR of YAP, leading to the downregulation of YAP along with its associated target genes, including cellular myelocytomatosis (c-Myc) and connective tissue growth factor (CTGF), thereby inhibiting proliferation in LC cells (78). MiR-506 has been shown to trigger apoptosis in HCC cells. Deng et al. demonstrated through experiments that miR-506 restrains HCC cell proliferation *in vitro* and tumor growth *in vivo*. Rho-associated protein kinase 1 (ROCK1), a target that miR-506 may directly target in HCC cells, has been shown to have an inverse relationship with miR-506-3p expression levels in HCC tissue. Furthermore, miR-506 is capable of causing cell cycle arrest and promoting apoptosis in cancer cells (15). miR-506 also affects the microenvironment around HCC cells; rapid cancer growth requires an adequate blood supply, and tumor vascular growth is essential for cancer development (79). HCC is generally considered a hypervascular tumor because it exhibits arterial enhancement compared with normal liver tissue (74). Sphingosine kinase 1 (SPHK1) facilitates cell survival, proliferation, transformation, and angiogenesis, while miR-506 targets SPHK1 to reduce its expression at the cellular, mRNA, and protein levels, inhibiting liver cancer angiogenesis (80). Previous studies show that miR-506 can directly target specific proteins to inhibit the growth of HCC cells, such as interleukin-8 (IL-8) (81) and STAT3 (82). Moreover, prior research has demonstrated that the functions of Ras homolog A (RhoA) in cancer cell migration, apoptosis, and proliferation are critical (83). The ROCK family contributes to cancer advancement by modulating cell growth and migration processes (84). miR-506 can modulate LC cell proliferation and apoptosis by influencing the RhoA/ROCK signaling cascade (85). Additional research demonstrates that down-regulating the expression of sirtuin1 (SIRT1) may prevent cancer cells from invasively growing and metastasizing with the combined treatment of miR-124-3p and miR-506-3p, thereby delaying the development of LC (86). Summarizing the evidence, there is extensive linkage between LC and miR-506, suggesting t that miR-506 may serve as a promising therapeutic target for managing LC.

### 3.3 miR-506 and osteosarcoma

Osteosarcoma (OS) is a highly malignant bone tumor predominantly affecting adolescents, with approximately 75% of patients aged 15 to 25 years (87). Characterized by a tendency for early metastasis and poor prognosis, OS poses a significant threat to adolescent health, and searching for new and effective therapeutic targets is an urgent priority. A growing body of research indicates that miR-506 functions as an inhibitor of OS initiation and progression through the modulation of various target genes, and elevated levels of its expression generally correlate with improved patient survival rates (55, 88, 89). miR-506 typically exerts inhibitory effects on OS. Yu et al. demonstrated through clinical sample studies and cell experiments that an increase in miR-506 levels can reduce the expression of Snail2 (55). This transcriptional repressor can promote EMT and, consequently, accelerate the metastasis of cancer cells (90). Additionally, astrocyte elevated gene-1 (AGE-1) has been confirmed as a downstream effector of miR-506; this microRNA can suppress AGE-1 expression via the wingless-type MMTV integration site family/beta-catenin (Wnt/β-catenin) signaling pathway to exert its inhibitory effect on OS cells, which was validated in an OS mouse model (89). Further studies have also pointed out that miR-506 impedes the aggressive behavior of OS by targeting Ras-related protein Rab-3D (RAB3D) (91), S-phase kinase-associated protein 2 (Skp2) (92), or SPHK1 (88). Similarly, the NF-κB signaling pathway is significant in various diseases, including cancer; IL-1β can reduce the expression of miR-506 through this pathway, leading to increased Jagged 1 (JAG1) expression and exacerbating the progression of OS (93). However, most clinical samples related to OS research have not been conducted in specific experiments based on different tumor stages. Moreover, many studies on miR-506 are still at the experimental stage and lack extensive clinical validation. These limitations challenge the clinical application of miR-506 in diagnosis and treatment.

### 3.4 miR-506 and colorectal cancer

Globally, CRC is categorized as the third most frequently occurring malignant neoplasm and accounts for the second-highest number of cancer-related fatalities (94). Researchers have identified that several genes targeted by miR-506 impact the biological behavior of CRC, including EZH2, which is implicated in carcinogenesis as an oncogene (95). Research by Zhang et al. confirmed that miR-506 is significantly downregulated in CRC tissues and cells through both *in vivo* and *in vitro* studies. By directly targeting the 3' UTR of EZH2, miR-506 reduces its level and subsequently exerts an anti-tumor response mediated by the Wnt/β-catenin signaling pathway (96). Ten-eleven translocation (TET) is highly expressed in CRC; miRNA-506 targets TETs and regulates the levels of TET1, TET2, and TET3, ultimately inhibiting the proliferation and invasion of CRC cells *in vivo* and *in vitro* (97). Furthermore, overexpression of miR-506 has been shown to inhibit cancer cell viability, invasion, and colony formation ability of CRC cells; moreover, miR-506 also targets laminin subunit gamma-1 (LAMC1), which is an important extracellular matrix (ECM) regulator to slow down cell migration and then reduce the metastasis of cancer cells (98). Other studies have similarly suggested a suppressive role of miR-506-3p in CRC, such as its targeting of cystatin-p1 (CSTP1) (30) and EZH2 (99). Combination therapy is also an effective treatment; miR-124 and miR-506 jointly target DNA (cytosine-5)-methyltransferase 3B (DNMT3B) and dynamin-1 (DNM1) and reduce global DNA methylation to reduce the effect of CRC (100). The sensitivity of radiotherapy and chemotherapy also reflects the inhibitory effect of miR-506 on CRC. Studies have shown that, based on the novel network-based method, radiosensitive patients exhibit significantly higher expression levels of hsa-miR-506-3p than radioresistant patients, offering a new foundation for future CRC treatment (101). Early cancer diagnosis, timely surgical intervention, and effective disease control are crucial. It has been found that miR-506 is helpful for early diagnosis of CRC (102). Although miR-506 shows significant anti-cancer effects, numerous genetic factors and signaling cascades contribute to CRC onset and progression, so the single-target action of miR-506 may not fully control cancer progression. Further research is essential to uncover the precise mechanisms and specific gene targets through which miR-506 influences CRC.

### 3.5 miR-506 and ovarian cancer

The incidence of OC is notably high among women. A significant proportion of patients are diagnosed at an advanced stage of the disease, leading to high mortality (103). In the past few years, several miR-506 target genes affecting the biological behavior of OC have been identified. Sun et al. observed a relationship between miR-506 expression and increased levels of E-cadherin (E-cad), along with the downregulation of vimentin (VIM), N-cadherin (N-cad), and snail homolog 2 (SNAI2). miR-506 can bind to the specific binding site of VIM, affecting the migration and drug resistance of epithelial ovarian cancer (EOC). These findings were confirmed by studies on clinical samples and animal experiments (104). Another study shows that miR-506-3p can target myotubularin-related protein 6 (MTMR6) to prevent cancer cells from proliferating in OC tissues, arresting the cells in the G0/G1 phase and thereby inducing apoptosis (17). A similar study indicates that miR-506 can target EZH2, reducing its expression and inhibiting OC (95). Other studies have also confirmed this conclusion (105). The CDK4/6-forkhead box m1 (FOXM1) pathway is a crucial signal network frequently activated in high-grade serous OC cases (106). Liu et al. demonstrated that miR-506 might suppress this pathway through *in vivo* and *in vitro* studies and clinical sample investigations, induce cancer cell apoptosis, and inhibit tumor progression in OC (107). Sirtuin1 (SIRT1), a deacetylation-regulating intracellular protein, activates tumor suppressor protein p53 to control tumor growth (108). In OC, miR-506-3p has been observed to interact with the 3'UTR of SIRT1 and reduce its expression, while studies confirm that elevated expression of SIRT1 promotes the proliferation of OC cells and inhibits apoptosis (109). Through integrated analyses, Yang et al. observed that miR-506 may be regulated in serous OC. Further, they confirmed at the cellular level that miR-506 can target SNAI2, a transcriptional repressor of E-cadherin, blocking EMT induced by transforming growth factor β (TGF-β). miR-506 enhances the expression of E-cadherin, reduces the expression of SNAI2 and VIM, and inhibits cancer cell migration and invasion (110). Research has also reported that miR-506 can directly target RAD51, reduce the expression of RAD51, regulate PARP-induced DNA damage repair, and improve chemosensitivity to cisplatin (111). Ultimately, the beneficial effects miR-506 on OC are extensive, encompassing inhibition of cancer cell migration, promotion of apoptosis, and enhancement of chemosensitivity, providing a solid foundation for future OC treatments.

### 3.6 miR-506 and other types of cancer

**Figure 2:**
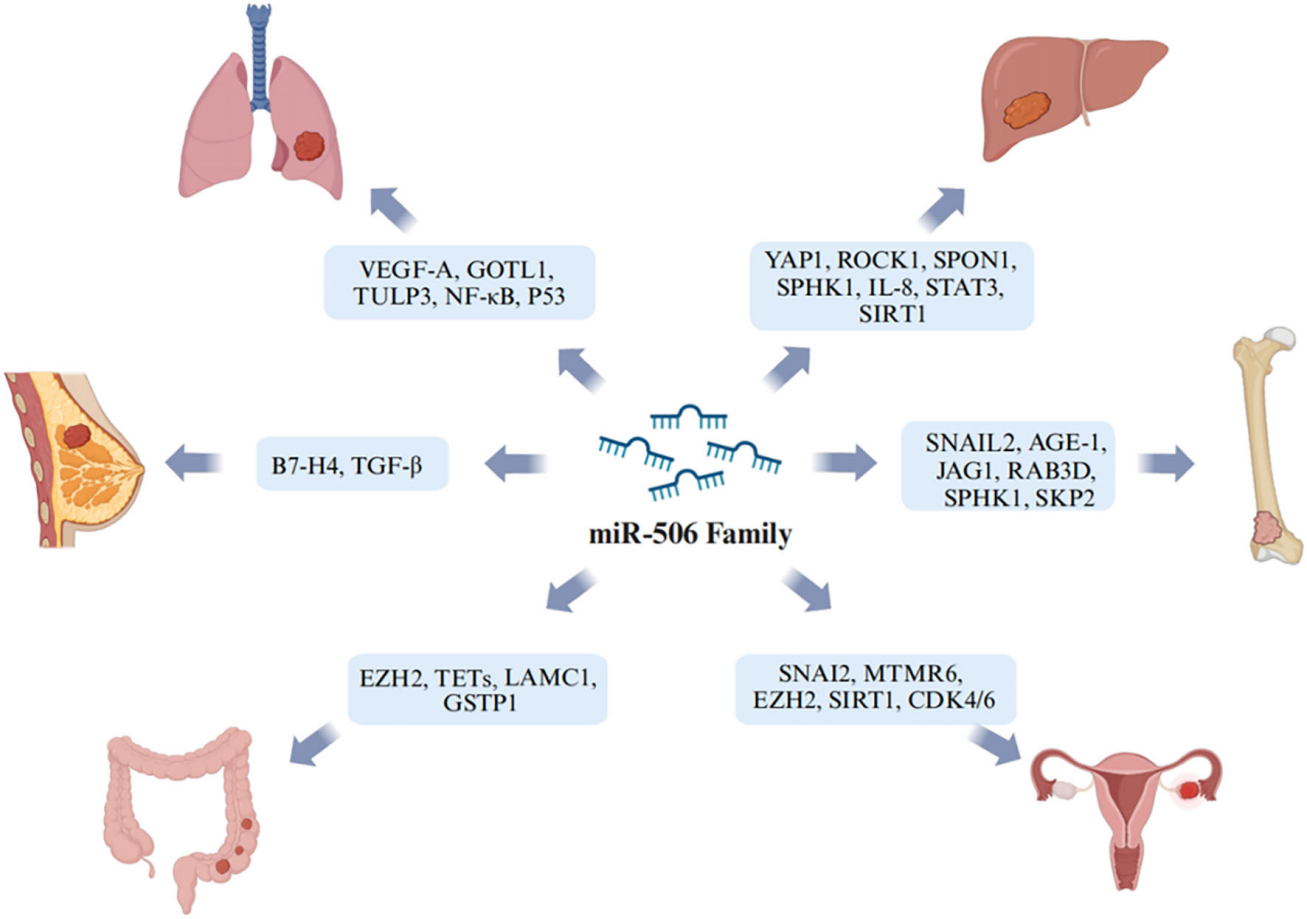
Schematic representation of the potential biological mechanisms through which miR-506 regulates various types of cancer. Image created with BioRender.com, with permission.

he impact of miR-506 on various cancer types has become increasingly recognized in the scientific community. The mechanisms of action and associated target genes of the miR-506 family in common cancers are illustrated in **Figure 2**. In particular, miR-506-3p has been shown to suppress the proliferation of papillary thyroid cancer (PTC) cells by targeting YAP1 (56). YAP1 is a known promoter of tumor progression (112) and acts as a carcinogen (113). YAP1 is the direct target gene of miR-506-3p, which targets YAP1 3’UTRs to down-regulate YAP1, allowing exposure of the CDK2/Cyclin E1 complex. Consequently, miR-506-3p is believed to inhibit the proliferation of PTC by suppressing YAP1 and modulating the YAP1-CDK2/Cyclin protein complex, which is involved in cell cycle control (56). YAP1 is an important target gene for many malignant cancers, such as HCC (78), papillary thyroid cancer (56), GC (114), and esophageal squamous cell carcinoma (ESCC) (115). In ESCC, YAP activates its downstream target SOX9 through TEA domain transcription factor 1 (TEAD1)-mediated binding transcription, targeting YAP’s miRNA, including miR-506-3p. This results in SOX9 inducing miR-506-3p, inhibiting YAP expression post-transcriptionally, creating a negative feedback mechanism (115). Other studies show that miR-506 functions as an oncosuppressive microRNA in nasopharyngeal carcinoma, exerting its tumor-suppressing activity primarily by suppressing Forkhead box protein Q1 (FOXQ1) expression (116). Similarly, in cervical cancer tissues, a reduction in miR-506 levels has been observed (117), which is negatively correlated with FOXQ1 expression. High expression of FOXQ1 enhances the migration and metastatic abilities of cervical cancer cells, promotes epithelial-mesenchymal transition (EMT), and decreases chemosensitivity. Based on these findings, there may be implications for developing treatment strategies for cervical cancer (118). Gli3 is a hedgehog pathway transcription factor. Wen et al. found that in cervical cancer, miR-506 can target Gil3, arrest cell cycle progression at the G1/S phase, curb proliferation, and induce apoptosis (119). Studies have shown that in prostate cancer cell lines (DU145 and PC-3), miR-506-3p expression is decreased, and its high expression inhibits tumor progression *in vitro*. At the same time, increased levels of N-acetylgalactosaminyltransferase 4 (GALNT4) negate the inhibitory effect of miR-506-3p on tumors (120). In addition, miR-506 targets ROCK1 and downregulates its expression, inhibiting neuroblastoma tumor growth (121). The association between BC survival and EMT has been widely confirmed in numerous studies, where miR-506 is shown to attenuate BC progression by regulating the EMT process (122, 123).

In cancer, the miR-506 family usually plays an inhibitory role; however, some research indicates that miR-506 may facilitate the progression of certain cancers. The miR-506-514 cluster is a collection of microRNAs on chromosome chrXq27.3, with miR-506-3p as its most prominent member (13). UM is the most prevalent primary intraocular malignancy in adults (124). It originates from melanocytes in the uveal tract, specifically in the choroid, iris, or ciliary body (125). Research suggests that the miR-506-514 cluster functions as a tumor suppressor in UM. Falzone et al. conducted a clinical sample study and divided the patients into T3-T4 vs. T1-T2, categorizing them as high-grade and low-grade groups. Compared with low-grade or surviving UM patients, the expression of mRNAs within the miRNA-506-514 cluster was significantly reduced in high-grade or deceased individuals (126). Nevertheless, findings from other relevant research studies indicate otherwise. Streicher et al. observed overexpression of the miR-506-514 cluster in melanoma patient tissues and cell lines, including SKMEL-2, SKMEL-5, A375, MALME-3M, and RPMI-7951. Inhibition of this cluster was shown to induce apoptosis in melanoma cells. Moreover, the sub-cluster effect was more evident than the whole cluster's (14). In addition, the role of miR-506 seems to be different between primary UM and metastatic melanoma. Specifically, miR-506 levels were elevated in patients with metastatic UM compared to those with primary UM, suggesting its potential as an early biomarker in disease progression (127). Melanoma is a biologically distinct malignancy that exhibits a strong response to immunotherapy, unlike most other solid tumors (128). Additionally, the anterior segment of the eye, particularly the iris, is more exposed to ultraviolet (UV) radiation, leading to DNA damage, distinguishing UM from other cancers (129). Tong et al. (26) reported that miR-506 is associated with chemoresistance in CRC. In the SW1161 CRC cell line, miR-506 induces resistance to hydroxy camptothecin by suppressing the expression of peroxisome proliferator-activated receptor alpha (PPARα), thereby reducing the drug’s efficacy. miR-506 can function as both a tumor suppressor and an oncogene in different cancer types, with several key factors contributing to this dual role. miR-506 exhibits tissue-specific expression patterns, which may determine whether it acts as a tumor suppressor or an oncogene. miR-506 is regulated by ceRNA networks, including lncRNAs and circRNAs, which act as "sponges" to modulate its availability (47). Its functional accessibility may vary significantly across different cancer types. Additionally, the tumor microenvironment, including immune infiltration, hypoxia, and stromal interactions, further regulates miR-506 activity, altering its downstream effects (28). Genetic variation is another major factor contributing to this paradox, encompassing structural genetic alterations such as chromosomal deletions/amplifications and mutations (130), defects in miRNA biogenesis mechanisms, and epigenetic changes, such as altered DNA methylation (131), which vary across different cancer types. Furthermore, no universally accepted protocol for miRNA sample collection, processing, detection, quantification, or therapeutic application leads to inconsistencies in research findings across different laboratories. As mentioned above, the role of the miR-506 family across various cancer types should be considered more comprehensively. Including more clinical samples and conducting more detailed cell and animal experiments would make experimental results more reliable. More specific studies should be performed according to different cancer types. Although the roles miR-506 in cancer have been partially identified, its exact across various cancer types remains incompletely understood.

## 4 miR-506 and drug resistance

Chemotherapy is a primary therapeutic method for the treatment of cancer, typically used as a monotherapy or in combination with surgery or radiation therapy to treat cancer patients (132). Chemotherapy mainly includes classic cytotoxic and molecular-targeted drugs (133), but drug resistance constantly challenges their efficacy. miRNAs play a significant role not only in cancer progression but also in modulating resistance to anticancer drugs. Since 2013, Blower et al. have elucidated the role of miRNAs in modulating chemotherapeutic responsiveness and resistance (134). Subsequent investigations have revealed that miRNAs can counteract chemoresistance by modulating genes implicated in drug resistance, optimizing drug targets, inhibiting cell migration, inducing cell apoptosis, and regulating the tumor microenvironment (135–137). As a microRNA with intricate regulatory functions, miR-506 is a key modulator in counteracting resistance to anticancer therapies. miR-506 is generally downregulated in drug-resistant tumors, whereas its upregulation has been shown to enhance tumor cell sensitivity to chemotherapeutic agents (138). The ability of miR-506 to sensitize drug-resistant cancer cells to chemotherapy is multifaceted, involving the regulation of cell cycle progression, apoptosis, drug transport, DNA repair, and EMT (139). Notably, the interaction between ceRNAs and miR-506 is pivotal in this process.

EMT is a critical biological process in which epithelial cells undergo phenotypic transition, characterized by the loss of cell-cell adhesion and the acquisition of mesenchymal traits, resulting in increased motility and invasiveness of cancer cells (140). In addition to its role in tumor progression, EMT is also implicated in chemoresistance (136, 141). In OC, miR-506 suppresses SNAI2-mediated EMT, thereby restricting the acquisition of stem-like properties in cancer cells and enhancing their response to chemotherapy and radiotherapy (111). Other studies have shown that the Wnt/β-catenin pathway, crucial in EMT and associated with cisplatin resistance, is targeted by miR-506 to suppress EMT and improve OC's susceptibility to PARP inhibitors and cisplatin (142). Overexpression of miR-506-3p in erlotinib-resistant (ER) cells has been demonstrated to downregulate sonic hedgehog (SHH) signaling, thereby reversing EMT-mediated erlotinib resistance (143). However, ceRNAs function as competitive endogenous RNAs that sequester miR-506, attenuating its EMT-inhibitory effects in cancer cells. For instance, FOXD2-AS1 promotes glioma cell proliferation and EMT by sequestering miR-506-5p, thereby modulating the expression of EMT-related genes such as CDH1, CDH2, and VIM (21). Similarly, HOXA11-AS facilitates HCC progression and EMT by regulating the miR-506-3p/Slug axis (144). Additionally, NEAT1 drives chemoresistance to gemcitabine in pancreatic cancer (PC) cells by regulating ZEB2 expression through the miR-506-3p/ZEB2/EMT axis (137). Chemoresistant cancer cells often evade apoptosis, a critical mechanism for treatment efficacy. Silencing of circRNAATL2 diminishes the ceRNA effect on miR-506-3p, curbing paclitaxel (PTX)-resistant OC cell drug resistance and up-regulated miR-506 can also induce cancer cell apoptosis by targeting NFIB, thus delaying disease progression (145). In CRC, overexpression of miR-506 in HCT116-OxR cells inhibits the expression of multidrug resistance protein 1 (MDR1)/P-glycoprotein 1 (P-gp) by down-regulating the Wnt/β-catenin pathway, promoting apoptosis in drug-resistant cells and enhancing sensitivity to oxaliplatin (146). Moreover, miR-506 can increase the sensitivity of drug-resistant cells to drugs by interfering with DNA damage repair. Homologous recombination (HR) is a critical pathway for the repair of DNA double-strand breaks (DSBs), and its dysfunction has been closely associated with increased sensitivity to DNA-damaging agents. RAD51 and RAD17 are two key components of the HR machinery. Previous studies have demonstrated that miR-506 directly targets RAD51, thereby sensitizing cancer cells to DNA damage and significantly enhancing the sensitivity of serous ovarian cancer (SOC) cells to cisplatin and PARP inhibitors (111). Recent evidence suggests that the miR-506-3p/RAD17 axis impairs DNA damage sensing in epithelial ovarian cancer (EOC) cells, leading to G2/M cell cycle arrest. This disruption may force severely damaged mitotic cells to enter mitosis, ultimately triggering mitotic catastrophe, thereby restoring miR-506-3p-induced platinum chemosensitivity (147). Moreover, in certain cancers, miR-506 enhances the sensitivity of cancer cells to anticancer agents by modulating metabolic pathways. miR-506 can restore chemosensitivity to 5-fluorouracil (5-FU) in drug-resistant cancer cells by downregulating polypyrimidine tract-binding protein 1 (PTBP1) expression and suppressing glycolytic activity. However, lncRNA SNHG16 acts as a molecular sponge for miR-506, thereby attenuating its inhibitory effects on PTBP1 and glycolysis (148). Notably, combining miR-506 with other miRNAs may serve as a promising strategy to overcome drug resistance. For example, miR-124-3P and miR-506-3p together can target EZH2, enhancing the sensitivity of sorafenib-resistant thyroid cancer cells to drugs (138).

Radiotherapy remains a cornerstone of cancer treatment and, together with surgery and chemotherapy, constitutes one of the three fundamental pillars of oncologic therapy. Increasing evidence underscores the critical role of microRNA dysregulation in modulating tumor radiosensitivity (101). Studies have demonstrated that DGCR5 knockdown enhances the radiosensitivity of human laryngeal squamous cell carcinoma by upregulating miR-506 and suppressing Wnt signaling (149). In OC, miR-506 suppresses SNAI2-mediated EMT, thereby restricting the acquisition of stem-like properties in cancer cells and enhancing their response to chemotherapy and radiotherapy (111). Fei et al. observed that plasma levels of hsa-miR-506-3p and hsa-miR-140-5p were significantly higher in radiosensitive CRC patients than in radioresistant patients. Subsequent experiments confirmed that overexpression of hsa-miR-506-3p and hsa-miR-140-5p significantly reduced cell proliferation, survival, and clonogenic potential in CRC cells following radiation exposure (101). Overall, research on mir-506 in improving tumor radioresistance remains limited, and further studies are needed to explore its role. Nevertheless, it is undeniable that mir-506 holds great potential as a promising radiosensitizer to enhance the efficacy of radiotherapy.

Although miR-506 holds promise in reversing drug resistance in certain cancers, its efficacy may vary depending on the cancer type and individual patient. Additionally, tumor cells may develop mechanisms to counteract miR-506-mediated reversal of resistance. Further research is needed to gain a deeper understanding of these processes. **Table 3** presents an overview of the interplay between miR-506 and chemoresistance in cancer treatment.

**Table 3:** miR-506 and drug resistance.

Cancer type	ceRNA	miR-506	Targets	Drugs	Influence	Results	References
Ovarian cancer	circATL2, ↑	miR-506-3p, ↓	NFIB, ↑	PTX	inhibit cancer cell apoptosis inhibit cell cycle progression	Promote	(145)
		miR-506-3p, ↑	EZH2,↓ β-catenin, ↓	Cisplatin Olaparib	inhibit cancer cell EMT	Improve	(142)
		miR-506, ↑	RAD51, ↓	Cisplatin Olaparib	inhibit DNA repair	Improve	(111)
	XIST, ↑	miR-506-3p, ↓	FOXP1, ↑	Carboplatin	inhibit cancer cell apoptosis promote cancer cell proliferation and autophagy	Promote	(192)
Osteosarcoma		miR-506-3p, ↑	STAT3, ↓	Doxorubicin	inhibit cancer cell proliferation promote cancer cell apoptosis	Improve	(163)
Epithelial ovarian cancer		miR-506-3p, ↑	RAD17, ↓	Platinum	inhibit DNA repair inhibit cell cycle progression	Improve	(147)
Colon cancer		miR-506, ↑	PPARa, ↓	Hydroxycamptothecin	inhibit cancer cell apoptosis	Promote	(26)
Non−small cell lung cancer		miR-506-3p, ↑	YAP1, ↓	Gefitinib	promote cancer cell apoptosis	Improve	(209)
		miR-506, ↑	SHH, ↓	Erlotinib	inhibit cancer cell EMT, stemness and proliferation promote cancer cell apoptosis	Improve	(143)
Colorectal cancer		miR-506, ↑	DNMT1, ↓	Cisplatin 5-Fluorouracil	promote cancer cell apoptosis inhibit cancer cell proliferation and DNA repair	Improve	(100)
		miR-506, ↑	MDR1/P-gp, ↓	Oxaliplatin	promote cancer cell apoptosis inhibit cancer cell growth	Improve	(146)
		miR-506-3p, ↑		Radiotherapy	inhibit cancer cell proliferation, survival rate and colonality	Improve	(101)
Gastric cancer	SNHG16, ↑	miR-506-3p, ↓	PTBP1, ↑	5-Fluorouracil	inhibit cancer cell glycolysis	Promote	(148)
Pancreatic cancer		miR-506, ↑		Palbociclib	promote cancer cell apoptosis	Improve	(210)
	NEAT1, ↑	miR-506-3p, ↓	ZEB2, ↑	Gemcitabine	promote cancer cell EMT	Promote	(137)
Esophageal squamous cell carcinoma		miR-506-3p, ↓	YAP, ↑	Cisplatin	promote cancer stem cell- like properties and EMT	Improve	(137)
Lung adenocarcinoma		miR-506, ↑	ATAD2, ↓	Cisplatin - based hyperthermia	promote cancer cell apoptosis inhibit cancer cell proliferation, migration, and invasion	Improve	(211)
Glioblastoma		miR-506, ↑	ETS1, ↓	Temozolomide	promote cancer cell apoptosis inhibit cancer cell proliferation, migration, invasion and autophagy	Improve	(212)
Thyroid carcinoma		miR-506, ↑	EZH2, ↓	Sorafenib	inhibit cancer cell proliferation	Improve	(138)
Hepatocellular carcinoma	KCNQ1OT1, ↑	miR-506, ↓	PD-L1, ↑	Sorafenib	inhibit cancer cell apoptosis impair T-cell immune surveillance promote cancer cell migration and invasion	Improve	(213)
Laryngeal cancer	DGCR5, ↑	miR-506, ↓	Wnt pathway, ↑	Radiotherapy	promote cancer stem cell - like properties	Improve	(149)

## 5 Clinical translation of miR-506

Although the miR-506 family exhibits oncogenic or ambiguous roles in a few cancer types, miR-506 is among the most significantly downregulated miRNAs in various malignancies (68, 78, 99, 119). Both *in vitro* and *in vivo* studies have consistently demonstrated its potent tumor-suppressive effects, highlighting its potential as a promising candidate for clinical translation. We reviewed the literature to analyze the challenges and strategies in translational medicine for miRNA-506. miR-506 has shown the ability to enhance chemotherapy sensitivity, supporting its use with conventional therapies. Preclinical models of NSCLC have suggested that combining miR-506 with cisplatin or immune checkpoint inhibitors may improve therapeutic efficacy (16). Furthermore, liquid biopsy techniques detecting miR-506 in blood or exosomes could facilitate patient stratification for targeted clinical trials. Phase I/II trial design similar to NCT04285476, which analyzed miRNA profiles in thyroid carcinoma, could be employed to validate miR-506 as a predictive biomarker (150). Although no dedicated clinical trials for miR-506 have been completed, implementing other miRNAs in clinical settings offers valuable insights and potential precedents for future applications. For instance, MRG-106 (Cobomarsen) is currently in Phase I and II clinical trials for treating lymphoma and leukemia (151). MesomiR-1 has been tested in a Phase I clinical trial for mesothelioma patients (152). Miravirsen (miR-122 inhibitor) successfully reduced HCV replication in Phase II trials, highlighting TS-miRNAs' potential in oncology. By integrating innovative drug delivery methods, biomarker stratification, and lessons learned from existing miRNA therapeutics, miR-506 could transition from preclinical research to clinical application. Nevertheless, current studies are limited to some common cancers; even for target genes in these cancers, research on target proteins remains insufficient. Despite evidence of its involvement in regulating gene expression and cell function, a detailed understanding of the precise molecular mechanisms by which miR-506 operates remains limited. This hampers the development of targeted therapeutic strategies involving miR-506, underscoring the need for further research. However, a significant gap remains in understanding the role of miR-506 in drug resistance. Some studies have clarified that the gene can counteract drug resistance through particular target proteins. Still, the precise pathway of influence is inaccurate, which is what future research should focus on. Since EMT is an essential cancer cell phenotype contributing to drug resistance, EMT inhibitors like miR-506 could be employed as crucial components of chemotherapy or targeted treatment medications, enhancing the clinical outcomes of existing cancer therapies. Even though studies on the connection between miR-506, EMT, and cancer cell drug resistance have increased recently, much hasn't been done to date on using miR-506 as a target to reduce drug resistance. Thus, future studies should focus more on this issue. Moreover, several studies use animal or cell models without clinical samples, or the number of clinical samples is not large enough. Some studies neglect to conduct in-depth disease stage and grade analyses, leading to outcome discrepancies. These issues need to be considered in further studies in this area. Notably, the biological function and expression pattern miR-506 exhibit variability across distinct disease states, adding complexity to its potential therapeutic applications. The inconsistency effects observed in specific diseases may be attributed to various factors, including individual differences, disease subtypes, and tissue specificity.

Effective delivery remains a critical barrier due to miR-506’s instability and poor tissue specificity. Lipid nanoparticles successfully delivered miR-34a in clinical trials (NCT01829971), providing a template for miR-506 encapsulation (153). The gelatin nanosphere (GN) delivery system enables the sustained and controlled release of exogenous miR-506, effectively targeting PENK and inactivating the ERK/FOS signaling pathway, thereby suppressing the growth and metastasis of triple-negative breast cancer (TNBC) (31). The delivery of miR-506-3p encapsulated in DOPC nanoliposomes effectively inhibited tumor growth and significantly enhanced the therapeutic effects of olaparib and cisplatin in orthotopic ovarian cancer mouse models (111). Additionally, exosome-mediated delivery of miR-506-3p enhances tumor-targeting efficiency, reducing proliferation and increasing apoptosis in CRC cells through the downregulation of GSTP1 (30). As shown in preclinical breast cancer models, pH-sensitive polymeric nanoparticles could release miR-506 mimics selectively in acidic TME (154). Applying in situ-forming hydrogels in intratumoral drug delivery, including their advantages in reducing systemic toxicity, provides insights into developing clinical application pathways for miRNA-506 (155).

Future research should focus on several key areas to establish miR-506 as a viable therapeutic agent. First, large-scale clinical trials are needed to determine its safety and efficacy. Next, delivery methods must be optimized to ensure tumor-specific targeting. Additionally, biomarker-based screening strategies should be developed for patient selection. Finally, exploring combination therapy approaches will be essential to maximize its clinical utility.

## 6 Discussion

Evidence has demonstrated that miRNAs play dual roles in various human malignancies, functioning as tumor suppressors (TS-miRNAs) or oncogenic miRNAs (oncomiRs). These small regulatory RNAs are involved in cancer initiation and progression by modulating multiple signaling pathways and cellular functions (156). Due to this multifunctionality, miRNAs have emerged as promising therapeutic targets with immense clinical potential. Two principal miRNA-based therapeutic strategies have been developed for cancer treatment: inhibition of overexpressed oncomiRs and restoration of downregulated TS-miRNAs (29). Specifically, three major approaches can be employed to silence oncomiRs, including miRNA sponges, antagomiRs/antimiRs, and CRISPR/Cas9 gene-editing technology (28). They sequester or destroy endogenous intracellular miRNAs, thereby preventing their binding availability to target mRNA. For restoring the function of tumor suppressor miRNAs that are downregulated or lost in cancer cells, miRNA replacement therapy using synthetic miRNA mimics is a viable strategy (157).

Despite the growing enthusiasm for miRNA-based therapeutics, several key challenges must be addressed before miRNA therapies can become routine clinical treatments. First, miRNA therapy faces the pharmacokinetic challenges of poor stability, short half-life, and uneven distribution *in vivo*. Naked miRNAs are highly susceptible to nuclease degradation, making it difficult to maintain therapeutic concentrations. Additionally, miRNAs are rapidly cleared from circulation and may accumulate in non-target tissues such as the liver and kidneys, reducing therapeutic efficacy (158). Improvement strategies include chemical modifications (2' -O-methylation, nucleic acid modification) and carrier encapsulation (lipid nanoparticles, polymer nanoparticles) to improve stability and targeting (28). The second is the challenge of off-target effects and targeted delivery. While offering therapeutic potential, miRNAs' extensive gene regulatory network also increases the risk of off-target effects that can disrupt normal cellular processes and induce unexpected biological effects. It has been shown that both single - and double-stranded oligonucleotides activate innate immune system responses (159) and can also be neurotoxic (160). Efficient and targeted delivery remains a significant hurdle. Viral vectors (e.g., AAV, lentivirus) offer high efficiency but raise concerns regarding immunogenicity and insertional mutagenesis. Non-viral delivery systems (e.g., lipid nanoparticles, exosomes) provide safer alternatives, but delivery efficiency, potential toxicity, and tissue specificity still need to be further optimized (28). Localized delivery strategies may offer an alternative approach. For example, Inoue et al. (161) demonstrated that a topical formulation containing miR-634 effectively suppressed tumor growth in two skin cancer models without systemic toxicity—however, only a limited number of cancers, such as primary and localized tumors. The third is the complexity of individualized therapy. miRNA expression varies significantly across cancer types, patient genetics, and tumor microenvironments, resulting in inter-patient variability in therapeutic response. Accurate identification of patients' miRNA expression profiles and the development of personalized miRNA therapy are key to future growth. In addition, combination treatment strategies (such as miRNA combined with targeted drugs or immunotherapy) may improve efficacy but still need to be validated in many clinical studies. Finally, there is no universally accepted protocol for miRNA detection, quantification, or therapeutic use, and an incomplete mass production and regulatory framework limits their clinical translation.

The current findings can further explore the molecular mechanisms by which miR-506 affects cancer cell growth. The infinite proliferation of cancer cells is driven by dysregulation of the cell cycle, and miR-506 plays a critical role in suppressing this process by targeting multiple cell cycle-related genes. Previous studies have demonstrated that miR-506 exerts its tumor-suppressive effects by directly targeting the 3′-UTR of CDK4/6, key regulators of the G1/S transition, thereby inducing G1 phase arrest and inhibiting cancer cell proliferation (72, 107). Furthermore, the combined application of miR-506 and miR-143 in the treatment of lung cancer LC and PC synergistically downregulates CDK1, CDK4, and CDK6, blocking both the G1/S and G_2_/M transitions and inducing robust apoptotic activity (71). One of the key mechanisms by which cancer cells evade immune surveillance is the suppression of apoptosis. miR-506 plays a crucial role in this process by regulating key apoptosis-related proteins. Apoptosis is tightly controlled by various genes and cytokines, with the Bcl-2 family serving as a central regulator. This family comprises two functionally opposing groups: anti-apoptotic proteins (e.g., Bcl-2, Bcl-xL) and pro-apoptotic proteins (e.g., Bax, Bak). These proteins govern mitochondrial membrane permeability and the subsequent release of cytochrome, ultimately determining whether a cell undergoes apoptosis (162). miR-506 influences the apoptotic process of cancer cells by regulating the expression of Bcl-2 family proteins in various cancer types. For instance, in NSCLC cell lines (16) and osteosarcoma cells (163), the overexpression of miR-506 significantly increases the sensitivity of cancer cells to apoptosis, which is closely associated with the upregulation of Bax and the downregulation of Bcl-2 at both the mRNA and protein levels. Additionally, studies have demonstrated that miR-506 can regulate NF-κB p65, specifically elevating reactive oxygen species (ROS) levels in tumor cells and activating the p53 pathway, thereby selectively inducing apoptosis in lung cancer cells (164). Moreover, miR-506 influences cancer cell fate by regulating other apoptosis-related genes. For instance, in Jurkat cells, a T-cell acute lymphoblastic leukemia (T-ALL) cell line, overexpression of miR-506 leads to decreased expression of pro-apoptotic genes such as p53 and p21 while increasing the expression of the anti-apoptotic gene Bcl-2 (165). This suggests that miR-506 may exhibit oncogenic properties in specific cancer types.

Cancer cells rely on DNA repair mechanisms to maintain genomic stability and survival. HR is a critical pathway for the repair of DNA double-strand breaks and is also associated with tumor chemoresistance. Studies have shown that miR-506 specifically targets the 3'-UTR of RAD51, thereby suppressing RAD51 gene expression, impairing the DNA damage response pathway, and enhancing chemosensitivity both *in vitro* and *in vivo*, ultimately reducing cancer cell growth (111, 166, 167). Furthermore, miR-506-3p has been identified to target RAD17, a key component of the HR pathway, thereby diminishing cancer cells' ability to sense DNA damage in OC. This leads to disrupting the G2/M cell cycle checkpoint, delaying G2/M cell cycle arrest, and potentially allowing severely DNA-damaged cells to enter mitosis, ultimately resulting in mitotic catastrophe (147). Aberrant human epidermal growth factor receptor-2 (HER2) signaling is implicated in various solid tumors, including BC, GC, biliary tract cancer, CRC, OC, and PC, where HER2 overexpression or amplification promotes tumor growth, invasion, and is associated with poor prognosis (168). Consequently, HER2 has been recognized as a key therapeutic target across multiple cancer types (169, 170). To date, no direct studies have demonstrated that miR-506 regulates HER2 expression or function. However, other miRNAs have been shown to modulate HER2. For instance, miR-101-5p (171), miR-489 (172), and miR-1226-3p (173) have been investigated for their roles in HER2-positive breast cancer. Given the significance of HER2 in tumor progression and treatment resistance, further research is warranted to explore the potential regulatory interactions between miR-506 and HER2, addressing this gap in the field.

Recent studies have highlighted the role of miR-506 in modulating tumor immune evasion and checkpoint regulation, suggesting its potential therapeutic significance. B7-H4, an immune checkpoint protein that negatively regulates T-cell activation, is overexpressed in BC and is associated with a poor immune response. It has been demonstrated that miR-506-3p downregulates the disease-associated rs10754339 "G" allele in B7-H4, thereby suppressing tumor progression (122). In addition, studies have shown that miR-506 promotes tumor immune escape by affecting programmed cell death-1 receptor (PD-1)/programmed cell death-ligand 1 (PD-L1) (174–176). As a potential regulator of immune checkpoint molecules, miR-506 has emerged as a promising candidate for combination therapies with immune checkpoint inhibitors (ICIs). Additionally, miR-506 reprograms tumor-associated macrophages (TAMs) from an immunosuppressive M2 phenotype to a pro-inflammatory M1 phenotype. This shift promotes cytotoxic T lymphocyte (CTL) infiltration, thereby reducing immune evasion in pancreatic ductal adenocarcinoma (PDAC) and strengthening immune-mediated tumor suppression (174).

Metabolic reprogramming, including increased glucose metabolism, fatty acid synthesis, and glutamine metabolism, is a major driver of cancer drug resistance. Among these, the enhancement of glycolysis is particularly critical (177). Tumor cells preferentially rely on glycolysis for energy production, even in the presence of sufficient oxygen, a phenomenon known as the “Warburg effect” (178). Studies have shown that enhanced glycolysis is closely associated with chemoresistance (179). For instance, PTBP1 promotes glycolysis by regulating the expression of pyruvate kinase M2 (PKM2), a key glycolytic enzyme, while miR-506-3p directly targets PTBP1, downregulating its expression and thereby reducing glycolysis, which restores 5-FU sensitivity in GC cells (148). Additionally, circHIPK3 binds to miR-506, suppressing its expression and subsequently upregulating pyruvate dehydrogenase kinase 2 (PDK2), leading to further activation of glycolysis and enhancing the proliferation and metastasis of HCC cells (180). Enolase 1 (ENO1), a key enzyme in glucose metabolism, is frequently overexpressed in various cancers and plays a crucial role in tumor progression (181). Beyond its role in glycolysis, ENO1 also interacts with choline kinase alpha (CHKα), a key enzyme in choline metabolism, stabilizing it and thereby promoting choline phospholipid metabolism and tumor cell proliferation (182). Dysregulated choline metabolism has been proposed as a novel cancer hallmark, with high CHKα expression being strongly associated with tumor progression and poor patient prognosis (182, 183). Furthermore, key glycolytic enzymes and substrates, including hexokinase 2 (HK2) (184), phosphoglycerate mutase 1 (PGAM1) (185), glucose transporters (GLUTs) (186), and lactate dehydrogenase (LDH) (187), are upregulated in various drug-resistant cancer cells, further supporting the critical role of glycolysis in chemoresistance. To date, limited studies have explored how miR-506 modulates metabolic pathways to overcome drug resistance, highlighting a promising avenue for future research

Continued research on miR-506 could provide essential insights into its complex mechanisms in different cancer types, providing significant clinical implications. miR-506 is down-regulated in a variety of cancers, including HCC (57), NSCLC (52), CC (59), and BC (123), and its expression is associated with tumor progression, drug resistance, and prognosis. Therefore, miR-506 can be a biomarker for cancer diagnosis and prognosis assessment (12). For example, in ovarian cancer, patients with low expression of miR-506 had poorer chemotherapy response (104), suggesting that miR-506 could be used to predict chemotherapy sensitivity and guide personalized treatment strategies. miR-506 regulates chemoresistance by modulating EMT, cell cycle progression, apoptosis, DNA damage repair pathways (e.g., RAD17), and key oncogenic signaling pathways (e.g., SHH, WNT/β-catenin) (139). Upregulating miR-506 expression through miRNA mimics or targeted therapeutic strategies may help reverse drug resistance and enhance chemotherapy efficacy, particularly for platinum-based compounds, doxorubicin, and EGFR-TKIs, ultimately improving patient outcomes (111, 142). Given its ability to suppress cancer cell proliferation and promote apoptosis, miR-506 represents a promising therapeutic target for miRNA-based gene therapy. Furthermore, lipid nanoparticles and viral vectors offer potential strategies for targeted miR-506 delivery to tumor tissues, presenting a novel and promising anticancer approach (12, 30, 139). miRNA has unique versatility and unique advantages in cancer treatment. Among them, miR-506 has become a key regulatory molecule in cancer biology and shows good therapeutic potential. Although its clinical application is still early, we are confident in its vast application potential. Future research should promote the advancement of miR therapy, including miR-506, by conducting comprehensive molecular studies, optimizing targeted drug delivery strategies, and launching large-scale randomized clinical trials.
